# Diagnostic and predictive accuracy of anti-mullerian hormone for ovarian function after chemotherapy in premenopausal women with early breast cancer

**DOI:** 10.1007/s10549-021-06508-w

**Published:** 2022-01-08

**Authors:** Richard A. Anderson, Tom W. Kelsey, Anne Perdrix, Nathalie Olympios, Orianne Duhamel, Matteo Lambertini, Florian Clatot

**Affiliations:** 1 MRC Centre for Reproductive Health, Queens Medical Research Institute, University of Edinburgh, 47 Little France Crescent, Edinburgh, EH16 4TJ UK; 2 School of Computer Science, University of St Andrews, St Andrews, UK; 3 Department of Biopathology, Centre Henri Becquerel, Rouen, France; 4 Normandie Univ, UNIROUEN, Inserm U1245, Rouen, France; 5 Department of Medical Oncology, Centre Henri Becquerel, Rouen, France; 6 Department of Internal Medicine and Medical Specialties (DiMI), School of Medicine, University of Genova, Genoa, Italy; 7 Department of Medical Oncology, U.O.C. Clinica di Oncologia Medica, IRCCS Ospedale Policlinico San Martino, Genoa, Italy

**Keywords:** Breast cancer, Biomarker, Anti-mullerian hormone, Ovarian function, Predictive testing

## Abstract

**Purpose:**

Accurate diagnosis and prediction of loss of ovarian function after chemotherapy for premenopausal women with early breast cancer (eBC) is important for future fertility and clinical decisions regarding the need for subsequent adjuvant ovarian suppression. We have investigated the value of anti-mullerian hormone (AMH) as serum biomarker for this.

**Methods:**

AMH was measured in serial blood samples from 206 premenopausal women aged 40–45 years with eBC, before and at intervals after chemotherapy. The diagnostic accuracy of AMH for loss of ovarian function at 30 months after chemotherapy and the predictive value for that of AMH measurement at 6 months were analysed.

**Results:**

Undetectable AMH showed a high diagnostic accuracy for absent ovarian function at 30 months with AUROC 0.89 (96% CI 0.84–0.94, *P* < 0.0001). PPV of undetectable AMH at 6 months for a menopausal estradiol level at 30 months was 0.77. In multivariate analysis age, pre-treatment AMH and FSH, and taxane treatment were significant predictors, and combined with AMH at 6 months, gave AUROC of 0.90 (95% CI 0.86–0.94), with PPV 0.79 for loss of ovarian function at 30 months. Validation by random forest models with 30% data retained gave similar results.

**Conclusions:**

AMH is a reliable diagnostic test for lack of ovarian function after chemotherapy in women aged 40–45 with eBC. Early analysis of AMH after chemotherapy allows identification of women who will not recover ovarian function with good accuracy. These analyses will help inform treatment decisions regarding adjuvant endocrine therapy in women who were premenopausal before starting chemotherapy.

## Introduction

The treatment of early breast cancer (eBC) frequently includes multi-agent chemotherapy; adjuvant endocrine therapy is also widely used in case of hormone receptor-positive tumour (HR+) to suppress the effect of remaining estrogen production and reduce the risk of relapse [1]. Extensive research has demonstrated the superiority of aromatase inhibitors (AIs) over tamoxifen as adjuvant endocrine therapy for postmenopausal eBC [2]. While AIs alone are ineffective in premenopausal women [3, 4], when co-administered with a gonadotropin releasing-hormone agonist (GnRHa), they achieve therapeutically adequate suppression of serum estrogen levels [5]. The superiority of combining AI with ovarian suppression as adjuvant endocrine therapy in premenopausal women as compared to tamoxifen-based treatment has been recently demonstrated in the SOFT and TEXT trials [6] and in the HOBOE study [7].

Many women with eBC become amenorrhoic after chemotherapy, the proportion increasing with age [8, 9]. As some show variable recovery, which may take 2 years or occasionally longer [10, 11], the diagnosis of a permanent menopausal state is often difficult. However, many women will have permanent loss of ovarian function during or shortly after chemotherapy, and accurate early identification of these women might allow optimization and simplification of the choice of adjuvant endocrine therapy [12].

The measurement of anti-Müllerian hormone (AMH) has become established as the most reliable biomarker of the number of small growing follicles in the ovary, which indirectly reflects the number of primordial follicles, i.e. the ovarian reserve [13]. AMH levels decline to undetectable at the time of the menopause [14, 15]. A substantial body of evidence has demonstrated that AMH levels fall in women during chemotherapy, with variable recovery depending on the treatment regimen [16–20], pre-treatment AMH levels and younger age [21–25], and possibly BMI [26]. Post-chemotherapy AMH measurement also predicts of ovarian function recovery [27–29]: if a woman with eBC has a very low or undetectable AMH level after chemotherapy, there is high confidence that she is indeed permanently menopausal [28]. Assessment shortly after completion of chemotherapy would aid clinical management; measurement of AMH shortly after completion of chemotherapy showed good prediction of women who would have ovarian failure at 24 months after diagnosis [27].

In this study, we investigated whether AMH measurement is a reliable method of identifying whether or not there is residual ovarian function following completion of chemotherapy in women aged 40 and over with eBC. This would potentially allow avoidance of unnecessary administration of GnRHa treatment as adjuvant endocrine therapy, with significant benefits in cost savings and in convenience to the patient.

## Methods

### Patients

This study was conducted within a cohort of consecutive patients with eBC diagnosed between 40 and 45 years of age who underwent (neo)adjuvant chemotherapy between January 2008 and December 2016 at the Henri Becquerel Cancer Center (Rouen, France). Of a total of 494 patients of appropriate age during that period, only patients with available stored blood samples before and at 6, 18 and 30 months after chemotherapy were included, and hormone assays were then performed. Chemotherapy was based on epirubicin, cyclophosphamide +/− a taxane (docetaxel in the great majority). Adjuvant endocrine therapy consisted of tamoxifen exclusively, with no exposure to AIs or GnRH agonists.

All patients gave written informed consent allowing the conservation and study of their biological samples. The present study was approved by the Institutional Scientific and Ethics Committees of Henri Becquerel Centre (registering order N°1917B).

### Hormone analyses

AMH, follicle stimulating hormone (FSH) and estradiol were measured in plasma using an electrochemiluminescence autoanalyser (Elecsys® assay reagents, cobas e601 analyser, Roche Diagnostics). All samples from the same patient were analysed in the same run to minimize between-run variability. For AMH (Elecsys AMH Plus), the limit of detection (LOD) is 0.01 ng/mL (0.07 pmol/L) and limit of quantification (LOQ) 0.03 ng/L. The between-run variability was 1.13% at 0.929 ng/mL, and 1.3% at 4.86 ng/mL. The estradiol assay (Elecsys Estradiol III) LOD is 18.4 pmol/L (5 pg/mL) and LOQ is 91.8 pmol/L. The FSH assay (Elecsys FSH) LOD is < 0.1 IU/L.

### Statistical methods

Data are presented as median and 95% confidence interval (CI). Changes in hormone concentrations over time were analysed by repeat measures ANOVA with Bonferroni correction for multiple comparisons. Receiver-operator characteristic (ROC) curve analyses were performed, reporting area under the curve (AUROC). Univariate analysis investigated simple relationships between detectable and undetectable AMH as binary category and later ovarian function as a binary category defined by a threshold level.

Multivariate analysis was also performed to assess the predictive performance of baseline and treatment characteristics (endocrine and non-endocrine) and post-chemotherapy endocrine factors in terms of later ovarian function. Multivariate analysis was performed in three stages. First, individual variables were assessed for prediction of undetectable AMH at 30 months’ post-treatment. Second, suitable candidate variables from the first stage were used in multivariate linear regression models (PRISM version 9, GraphPad Software LLC, San Diego USA) to provide estimates of AUROC, PPV and NPV. Third, and to guard against the multivariate linear regression models over- or underfitting the data (i.e. supplying estimates that are unlikely to generalize to new data instances) a full machine learning workflow was performed using scikit-learn version 0.46 within Python version 3.9.2. The workflow stages were: shuffling and splitting data into 70% train and 30% validation subsets; fivefold cross-validated grid search of 420 options for optimal hyperparameters for the random forest algorithm applied to the test data; cross-validated application of the optimal model on the training data; application of the model to the validation data subset that mimics new data instances. A linear regression model was considered validated in terms of clinical utility if (a) the cross-validated test performance of the random forest model for the test data was close to the validation performance (i.e. the model is neither over- nor-underfitting the data), and (b) the validation AUROC is similar to the estimate found by linear regression.

## Results

Samples from a total of 206 women were analysed, with complete sample sets in 197 women. Most (76%) patients had HR+ disease and received tamoxifen; 48 patients had HR- tumours and did not receive adjuvant endocrine treatment. Chemotherapy regimens were based on 6 cycles of cyclophosphamide with an anthracycline, and the addition of a taxane in 84%; 22% received anti-HER2 targeted therapy. Six patients received 8 cycles of chemotherapy in the context of inflammatory breast carcinoma. Among the 173 patients exposed to a taxane, all but 3 were received docetaxel. Patient characteristics, tumour and treatment details are described in Table 1.

**Table 1:** Baseline characteristics of study population

Patients characteristics	All patients (*N* = 206)
Age at diagnosis, mean (SD)	42.76 (1.76)
Weight (kg), mean (SD)	67.54 (14.57)
Body mass index (kg/m^2^), mean (SD)	25.12 (5.29)
Smoker, *N* (%)	72 (35%)
Genetic mutation, *N* (%)	14 (6.8%)
BRCA1	9 (64%)
BRCA2	5 (36%)
Tumour characteristics, *N* (%)	
Histological grade	
Grade I	9 (4.4%)
Grade II	93 (45.1%)
Grade III	104 (50.5%)
Hormone Receptor positivity	162 (78.6%)
Oestrogen receptor	161 (78.2%)
Progesterone receptor	140 (68%)
HER2 positive	47 (22.8%)
Triple negative	32 (15.5%)
Pathological nodal status positivity	119 (57.8%)
Tumour size (T)	
T1	91 (44.2%)
T2	91 (44.2%)
T3	19 (9.2%)
T4a	1 (0.5%)
T4b	1 (0.5%)
T4d	3 (1.5%)
Surgical treatment, *N* (%)	
Conservative	128 (62.1%)
Mastectomy	78 (37.8%)
Adjuvant or neoadjuvant treatment, *N* (%)	
Radiation therapy	200 (97.1%)
Endocrine therapy	160 (77.7%)
Chemotherapy	206 (100%)
Chemotherapy regimen, *N* (%)	
With taxane	173 (84%)
Without taxane	33 (16%)
With HER2 inhibitor	46 (22%)
Fertility history, *N* (%)	
Pregnancy before treatment	182 (88.3%)

**Fig. 1:**
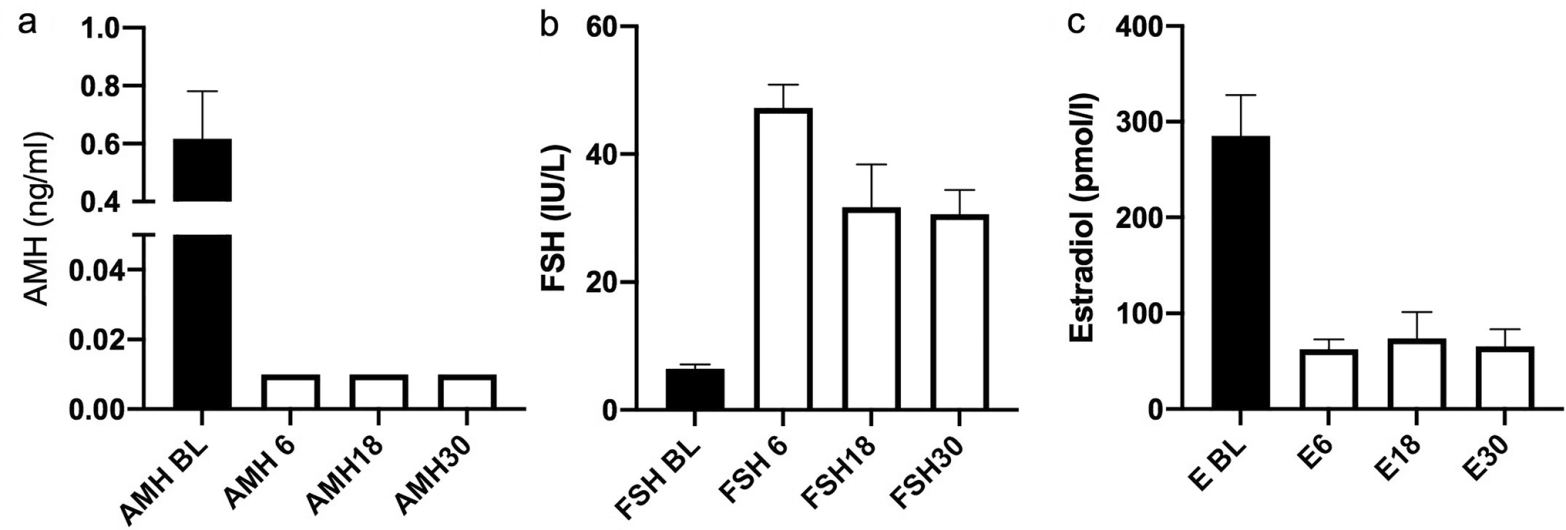
AMH (**a**), FSH (**b**) and estradiol (**c**) concentrations pre-treatment and at 6, 18 and 30 months after completing chemotherapy for eBC. Median ± 95% CI, *N* = 206

AMH concentrations fell markedly following chemotherapy from a median of 0.62 (IQR 0.21–1.31) ng/ml at baseline becoming undetectable in 137 (70%) of 197 women at 6 months (Fig. 1) and with very little recovery thereafter (*P* < 0.001 vs pre-treatment at all time points). AMH was undetectable in 115 (58%) and 119 (60%) women at 18 and 30 months respectively. There was a rise in FSH from 6.4 (IQR 0.2–1.3) IU/L at baseline to 30.6 (IQR 18.2–47.1) IU/L at 30 months (*P* < 0.001 vs baseline at all time points), and a sustained fall in estradiol from 285 (IQR 168–2095) pmol/L at baseline to 65.6 (IQR 45.2–4111) pmol/L at 30 months (Fig. 1b and c).

**Fig. 2:**
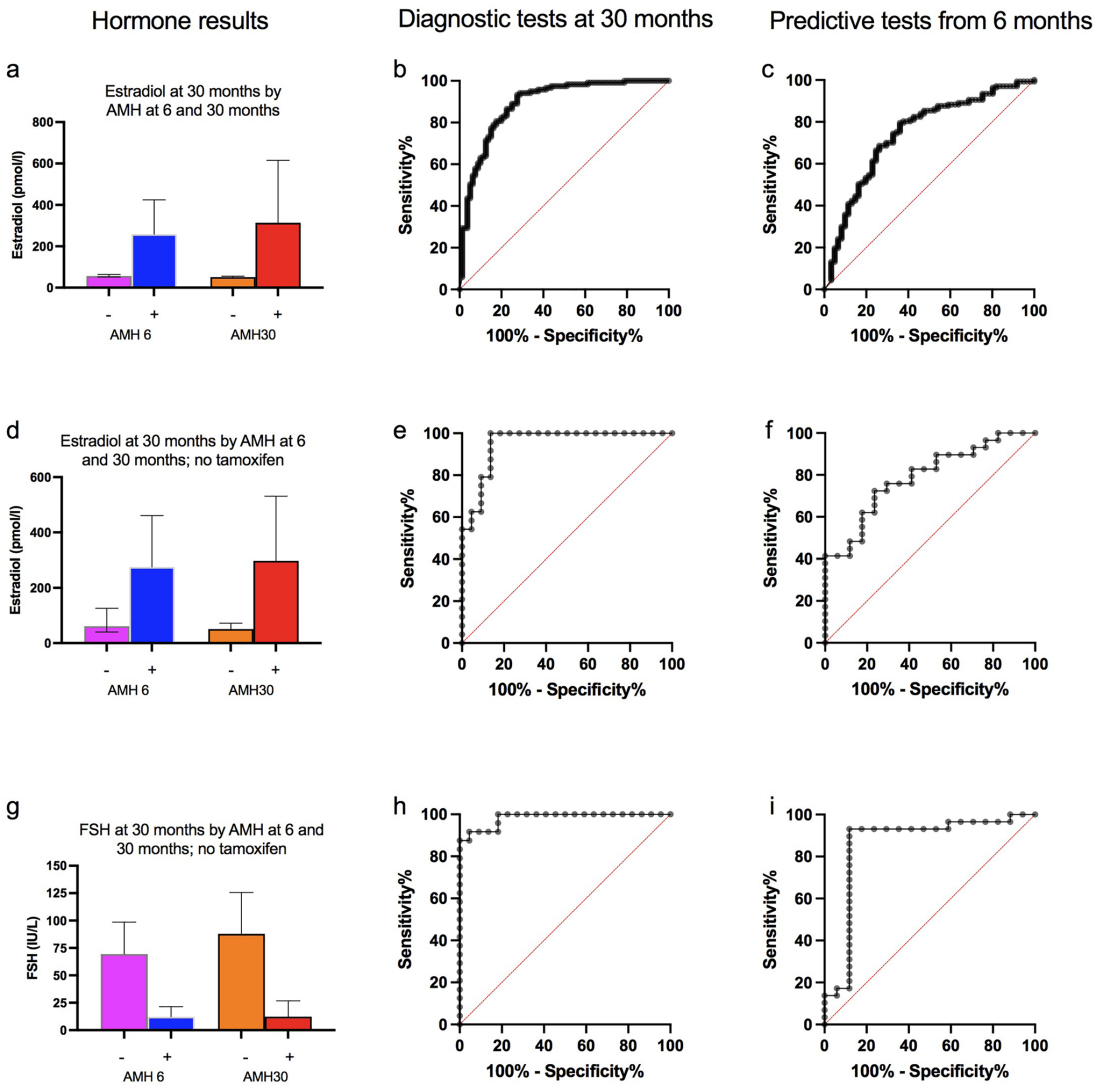
Hormone results (**a**, **d**, **g**), results of diagnostic testing at 30 months (**b**, **e**, **h**) and predictive testing of 30 months by data at 6 months (**c**, f**, i**). **a** Estradiol levels at 30 months by AMH at 6 and 30 months, divided into AMH undetectable (−) vs AMH detectable (+), with ROC curves for diagnostic analysis by AMH at 30 months (**b**), and prediction by AMH at 6 months (**c**). **d** In women not treated with tamoxifen: Estradiol levels at 30 months by AMH at 6 and 30 months, divided into AMH undetectable (−) vs AMH detectable (+), with ROC curves for diagnostic analysis by AMH at 30 months (**e**), and predictive analysis by AMH at 6 months (**f**). **g** In women not treated with tamoxifen: FSH levels at 30 months by AMH at 6 and 30 months, divided into AMH undetectable (−) vs AMH detectable (+), with ROC curves for diagnostic analysis by AMH at 30 months (**h**), and predictive analysis by AMH at 6 months (**i**)

The value of AMH as an index and as a predictor of absent ovarian function after recovery from chemotherapy was explored. At 30 months, women with undetectable AMH at that time (*n* = 119) had median estradiol of 50 pmol/L (IQR 34–68), whereas it was 313 pmol/L (IQR 102–1052) (*P* < 0.0001) in the 80 women with detectable AMH (Fig. 2a). Thus, undetectable AMH at 30 months showed a high diagnostic accuracy for absent ovarian function with AUROC 0.89 (96% CI 0.84–0.94, *P* < 0.0001; Fig. 2b), with peak likelihood ratio of 25.3 at an estradiol concentration of 38.1 pmol/L, with sensitivity 29% and specificity 98.8%. This demonstrated that after recovery from chemotherapy, undetectable AMH was an accurate diagnostic test of absent ovarian activity, and therefore it was used as an outcome measure for multivariate predictive analyses, supporting analysis of estradiol levels.

For prediction of later ovarian activity, women with undetectable AMH at 6 months (*N* = 137) had median estradiol levels at 30 months of 56 pmol/L (IQR 40–104), vs 258 pmol/L (IQR 69–780) (*P* < 0.0001) in women with detectable AMH at 6 months (*n* = 62) (Fig. 2a). AUROC for estradiol at 30 months by undetectable AMH at 6 months was 0.75 (95% CI 0.67–0.82, *P* < 0.0001; Fig. 2c), with sensitivity 19.7% and specificity 95.1% at estradiol concentration of 34.4 pmol/L, at which likelihood ratio peaked at 4.0. The positive predictive value of undetectable AMH at 6 months for a menopausal estradiol level (< 110 pmol/L [30]) at 30 months was 0.77. Supporting this, AMH at 6 months for prediction of undetectable AMH at 30 months was explored. AUROC was 0.76 (CI 0.68–0.83, *P* < 0.0001), with PPV of undetectable AMH at 6 months for unpredictable AMH at 30 months of 0.78.

As both FSH and estradiol may be impacted by tamoxifen treatment, data were additionally analysed separately in the 48 women not taking tamoxifen. AMH was undetectable in 29 (60%) of these women at 6 months, and also in 29 (60%) women at 30 months. At 30 months, median estradiol concentrations of women grouped by detectable vs undetectable AMH levels at both 30 and 6 months (Fig. 2d) were similar to those groups in the whole cohort of women (Fig. 2a). ROC analysis demonstrated the very high predictive value of undetectable vs detectable AMH at 30 months for estradiol at 30 months, AUROC 0.95 (0.89–1.00, *P* < 0.0001; Fig. 2e); similarly, AMH at 6 months was predictive of estradiol at 30 months, with AUROC 0.79 (0.66–0.92, *P* = 0.001) (Fig. 2f).

FSH is an established diagnostic test for POI, thus analyses were performed in women not taking tamoxifen for AMH as a predictor of FSH > 25 IU/L. At 30 months, median FSH in women with undetectable AMH at that time point was 87.8 IU/L (IQR 67.7–126.9) vs 12.4 IU/L (8.6–25.2) (*P* < 0.0001) in those with detectable AMH. Analysis by AMH at 6 months gave comparable results (Fig. 2g), with median FSH at 30 months of 69.4 IU/L (42.5–108.8) vs 12.2 IU/L (8.6–23.9). The diagnostic value was assessed by ROC analysis for AMH at 30 months, showing AUROC 0.98 (0.96–1.00), and for prediction by AMH at 6 months, AUROC was 0.86 (0.72–0.99) (both *P* < 0.0001; Fig. 2h and i) with peak likelihood ratio of 7.9 at FSH 27.7 IU/L. An undetectable AMH at 6 months had a PPV for FSH > 25 IU/L at 30 months of 0.93, indicating a very high predictive value for long-term POI after chemotherapy.

### Multivariate analyses

**Fig. 3:**
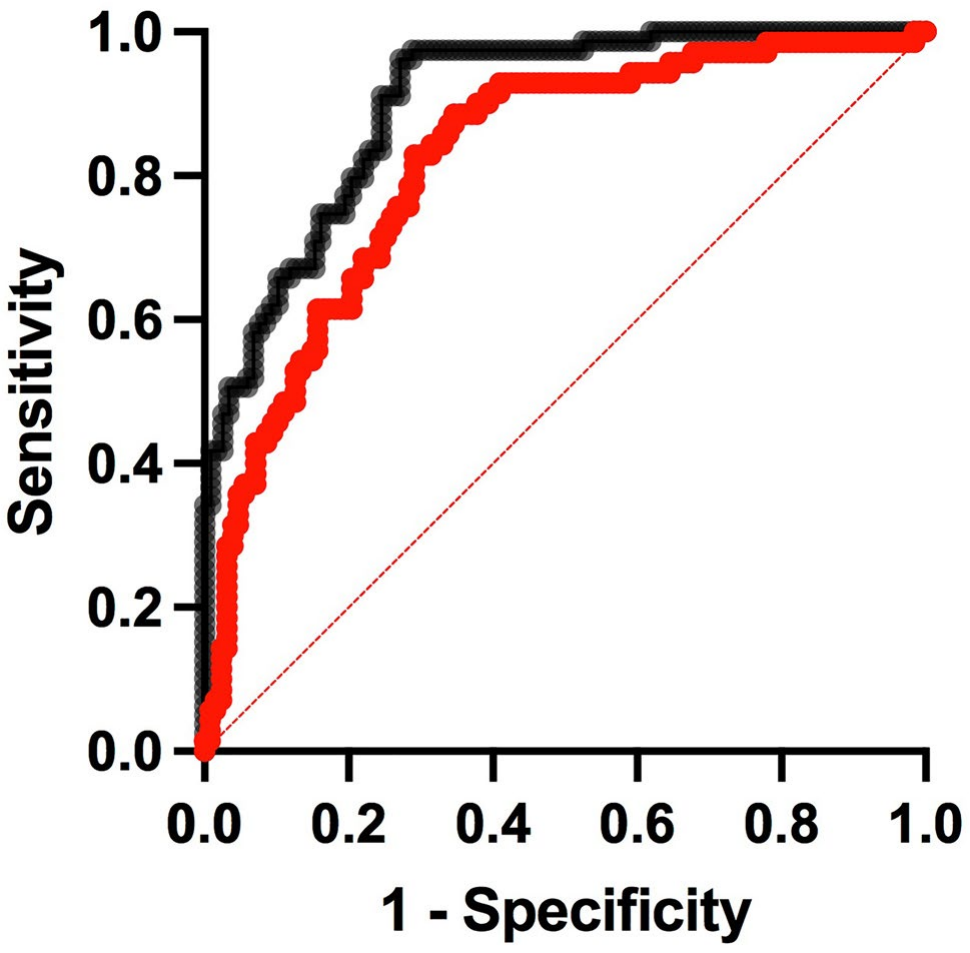
Multivariate ROC analysis: prediction of undetectable AMH (black) or estradiol < 110 pmol/L (red) at 30 months by AMH at 6 months, pre-treatment hormone data and taxane treatment

The variables age, pre-treatment AMH and FSH, and taxane treatment were found to be significant predictors of AMH at 30 months; BMI and pre-treatment estradiol were not (Table 2). The significant predictors were then combined with AMH at 6 months for prediction of AMH at 30 months (Table 2). This gave AUROC of 0.90 (95% CI 0.86–0.94), with PPV 0.79 and NPV 0.79 (Fig. 3). Using estradiol at 30 months of < 110 pmol/L as the outcome, the same variables gave AUROC of 0.82 (0.76–0.90), PPV 0.68 and NPV 0.76 (Fig. 3).

**Table 2:** Results of univariate, multivariate and random forests analysis

Univariate analysis vs AMH at 30 months							
Variable	OR	95% CI					
Taxane	0.70	0.52–0.96					
Age	0.99	0.98–0.99					
BMI	1.04	0.98–1.09					
BL AMH	6.84	3.89–13.41					
BL FSH	0.92	0.89–0.95					
BL estradiol	1	0.99–1.00					

Multivariate analysis vs AMH at 30 months				AUROC	95% CI	NPV	PPV
Age	0.97	0.94–1.01	Multivariate	0.90	0.86–0.94	79.4	78.8
Taxane	3.54	1.17–12.00	RF	0.89		71.9	81.6
BL AMH	4.16	2.36–8.25					
BL FSH	0.93	0.84–0.99					
AMH6	0.2	0.08–0.50					

Multivariate vs estradiol at 30 months < 110 pmol/L							
Age	0.98	0.95–1.00	Multivariate	0.82	0.76–0.88	75.5	68.0
Taxane	1.64	0.66–4.42	RF	0.76		66.8	82.6
BL AMH	2.03	1.45–3.01					
BL FSH	0.99	0.95–1.02					
AMH6	0.36	0.16–0.78					

If no pre-treatment hormone data vs AMH at 30 months							
Age	1.01	0.99–1.03	Multivariate	0.71	0.63–0.79	76.6	78.3
Taxane	3.69	1.36–11.06	RF	0.85		56.3	92.1
AMH6	0.07	0.03–0.15					

Pre-treatment data only							
Age	0.96	0.92–0.99	Multivariate	0.88	0.83–0.92	78.6	76.5
Taxane	2.2	0.80–6.66	RF	0.87		65.6	84.2
BL AMH	5.44	3.09–10.82					
BL FSH	0.91	0.82–0.98					

*AMH6* grouped by undetectable vs detectable AMH at 6 months, *BL* baseline (pre-treatment) sample, *RF* random forest, *OR* odds ratio, *AUROC* area under the curve of receiver-operator characteristic analysis *NPV* negative predictive value, *PPV* positive predictive value

Two additional analyses were performed to assess prediction if pre-treatment hormone data were not available, and of pre-treatment variables (including taxane treatment) only. In the absence of pre-treatment hormone data, age/taxane treatment/AMH6 gave AUROC 0.71 (0.63–0.79) with PPV 0.78 and NPV 0.77. Conversely, age/taxane treatment/pre-treatment hormone variables gave AUROC 0.88 (0.83–0.92), PPV 0.77 and NPV 0.79.

The linear regression models were validated by random forest models with data retained for validation purposes, with random forest AUROC within the 95% CI for the AUROC reported for the logistic regression model (Table 2). Without pre-treatment hormone data, the random forest AUROC was significantly higher at 0.85 compared to 0.71, indicating that the linear regression model is underfitting the data. For the other analyses, the cross-validated test accuracy of each optimal random forest model was within 4.6 percentage points of the validation accuracy.

## Discussion

Assessment of ovarian function after chemotherapy is critical for women with breast cancer where decisions about appropriate endocrine treatment are required [12]. Moreover, many women also want to know whether a later pregnancy might possible. There is increasing evidence for the value of AIs in women who are premenopausal at the time of diagnosis [6, 7], but if there is ovarian activity after chemotherapy, concomitant ovarian suppression with a GnRH agonist is necessary to ensure adequate suppression of estradiol levels. There is however uncertainty as to the degree of suppression of estradiol levels that is required and accuracy of immunoassays at these low concentrations [31], indicating a need for improved biomarkers of ovarian function.

In these analyses we have explored the potential accuracy of AMH as a biomarker of ovarian activity after chemotherapy for eBC, as a diagnostic test at 30 months after completion of chemotherapy (thus allowing for any recovery) and a predictive test at 6 months after chemotherapy. AMH levels fell dramatically after chemotherapy, with overall very little recovery thereafter, as previously reported [17–20]. Undetectable AMH at that time accurately distinguished women with low estradiol levels, indicating that AMH is a clinically useful index of ovarian function in this context. The best estradiol concentration cut-off distinguishing women with and without detectable AMH levels was 34 pmol/L, similar to the upper limit in postmenopausal women using mass spectroscopy [32].

While accurate diagnosis of absent ovarian function after allowing for potential recovery is of value, it would be of yet greater clinical value to be able to predict post-treatment menopausal status at the end of chemotherapy. At 6 months after chemotherapy, thus at a clinically relevant time point to decide on whether ovarian suppression might be necessary [6, 33], AMH levels were undetectable in 70% of the population. This had clear value in predicting later ovarian function, by estradiol levels or AMH at 30 months. Thus, women aged over 40 treated for eBC with anthracycline- and taxane-based chemotherapy regimens who have an undetectable AMH level at 6 months, using a highly sensitive assay, are very likely to show permanent loss of ovarian function, and ovarian suppression may not be required. This supports a previous analysis of a smaller group of women with eBC (*n* = 32), where undetectable AMH at the end of chemotherapy accurately predicted lack of recovery of ovarian function in women aged over 40, but not younger women [27].

However, some women did show a degree of recovery of ovarian function, mostly within 18 months of chemotherapy. This late recovery has been demonstrated previously [10, 11], and while more likely in younger women, the present analysis documents its prevalence in women aged 40—45 years at approximately 11% of the population studied. While the recovery in AMH levels was small, estradiol levels in some women were high, reflecting the effect of tamoxifen treatment inducing multifollicular ovarian activity.

While cut-off levels of estradiol for diagnosis of menopausal status are debated [31], there is consensus that the biochemical diagnosis of menopause or POI should be based on FSH levels, with high levels reflecting a lack of estrogen and inhibin-mediated feedback on the hypothalamus and anterior pituitary gland. A value of 25 IU/L is widely recommended for both POI and natural menopause [34–36], although others suggest a higher value. As tamoxifen, through estrogen receptor antagonism, raises FSH levels, this can only be used in women not taking any endocrine therapy. In that group of women, our study showed that undetectable AMH levels at both 6 and 30 months were associated with similar discrimination of estradiol levels as in the whole study population, and analysis of diagnostic accuracy showed slightly greater precision for both diagnosis at 30 months and prediction at 6 months of both 30-month estradiol and AMH than in the wider group. PPV of undetectable AMH at 6 months for elevated FSH consistent with a diagnosis of POI at 30 months was a remarkable 0.93.

While a single assay of AMH at 6 months provides good prediction of later ovarian function and has the clinical benefit of simplicity, we also explored whether additional endocrine, patient and treatment factors could improve this prediction. We and others have shown that pre-treatment AMH is predictive [21–25], as is age, with BMI also contributing in some studies [26]. The addition of a taxane to cyclophosphamide-based regimens also increases ovarian toxicity [17, 20]. In multivariate analysis, pre-treatment AMH and taxane treatment were the most important predictors: the limited value of age is likely to reflect the narrow age range in this specific study population. Including all identified factors resulted in PPV 0.79 for prediction of undetectable AMH at 30 months: random forest analysis gave a similar value of 0.82. Very similar results were obtained using estradiol at 30 months as the outcome variable. Analysis without pre-treatment hormone data gave similar results (though with an improvement in PPV to 0.92 by random forest), and by pre-treatment variables only (thus including pre-treatment AMH and taxane treatment) gave PPV of 0.77, with again better prediction by random forest analysis with PPV 0.84. Thus, using this approach with partial data retention for validation to prevent over-fitting allows accurate prediction of long-term ovarian function from either a single post-chemotherapy AMH test alone, or supplemented by knowledge of pre-treatment AMH and taxane treatment, or indeed with similar accuracy from pre-treatment AMH and taxane treatment alone. Therefore, this has validity and utility in a range of clinical scenarios, depending on which variables are known.

## Conclusion

These data demonstrate that in women aged 40–45 treated for eBC and after time to allow any recovery of ovarian function, an undetectable AMH level, using this assay platform, is a reliable diagnostic test for lack of ovarian function. Furthermore, early analysis of AMH after completion of chemotherapy allows identification of women who will not recover ovarian function with good accuracy. The combination of pre-treatment AMH measurement with knowledge of whether treatment will include a taxane in anthracycline/cyclophosphamide-based chemotherapy also provides good prediction of long-term ovarian function. These analyses will help inform treatment decisions regarding adjuvant endocrine therapy and the need for adding ovarian suppression to an AI in women who were premenopausal before starting chemotherapy.

## References

[1] Cardoso F, Kyriakides S, Ohno S, Penault-Llorca F, Poortmans P, Rubio IT, Zackrisson S, Senkus E (2019). Early breast cancer: ESMO clinical practice guidelines for diagnosis, treatment and follow-up. Ann Oncol.

[2] Dowsett M, Forbes JF, Bradley R, Ingle J, Aihara T, Bliss J, Boccardo F, Coates A, Coombes RC, Cuzick J, Dubsky P, Gnant M, Kaufmann M, Kilburn L, Perrone F, Rea D, Thurlimann B, van de Velde C, Pan H, Peto R, Davies C, Gray R, Early Breast Cancer Trialists’ Collaborative G (2015). Aromatase inhibitors versus tamoxifen in early breast cancer: patient-level meta-analysis of the randomised trials. Lancet.

[3] Guerrero A, Gavila J, Folkerd E, Ortiz B, Martinez F, Garcia A, Climent MA, Guillem V, Ruiz A (2013). Incidence and predictors of ovarian function recovery (OFR) in breast cancer (BC) patients with chemotherapy-induced amenorrhea (CIA) who switched from tamoxifen to exemestane. Ann Oncol.

[4] van Hellemond IEG, Vriens IJH, Peer PGM, Swinkels ACP, Smorenburg CH, Seynaeve CM, van der Sangen MJC, Kroep JR, de Graaf H, Honkoop AH, Erdkamp FLG, van den Berkmortel FWPJ, Kitzen JJEM, de Boer M, de Roos WK, Linn SC, Imholz ALT, Tjan-Heijnen VCG (2017). Ovarian function recovery during anastrozole in breast cancer patients with chemotherapy-induced ovarian function failure. JNCI J Natl Cancer Inst.

[5] Stein RC, Dowsett M, Hedley A, Gazet JC, Ford HT, Coombes RC (1990). The clinical and endocrine effects of 4-hydroxyandrostenedione alone and in combination with goserelin in premenopausal women with advanced breast cancer. Br J Cancer.

[6] Francis PA, Pagani O, Fleming GF, Walley BA, Colleoni M, Lang I, Gomez HL, Tondini C, Ciruelos E, Burstein HJ, Bonnefoi HR, Bellet M, Martino S, Geyer CE, Goetz MP, Stearns V, Pinotti G, Puglisi F, Spazzapan S, Climent MA, Pavesi L, Ruhstaller T, Davidson NE, Coleman R, Debled M, Buchholz S, Ingle JN, Winer EP, Maibach R, Rabaglio-Poretti M, Ruepp B, Di Leo A, Coates AS, Gelber RD, Goldhirsch A, Regan MM, Soft, Investigators T, The International Breast Cancer Study G (2018). Tailoring adjuvant endocrine therapy for premenopausal breast cancer. N Engl J Med.

[7] Perrone F, De Laurentiis M, De Placido S, Orditura M, Cinieri S, Riccardi F, Ribecco AS, Putzu C, Del Mastro L, Rossi E, Tinessa V, Mosconi AM, Nuzzo F, Di Rella F, Gravina A, Iodice G, Landi G, Pacilio C, Forestieri V, Lauria R, Fabbri A, Ibrahim T, De Maio E, Barni S, Gori S, Simeon V, Arenare L, Daniele G, Piccirillo MC, Normanno N, de Matteis A, Gallo C (2019). Adjuvant zoledronic acid and letrozole plus ovarian function suppression in premenopausal breast cancer: HOBOE phase 3 randomised trial. Eur J Cancer.

[8] Anderson RA, Amant F, Braat D, D’Angelo A, Chuva de Sousa Lopes SM, Demeestere I, Dwek S, Frith L, Lambertini M, Maslin C, Moura-Ramos M, Nogueira D, Rodriguez-Wallberg K, Vermeulen N, ESHRE Guideline Group on Female Fertility Preservation (2020). ESHRE guideline: female fertility preservation. Hum Reprod Open.

[9] Lambertini M, Peccatori FA, Demeestere I, Amant F, Wyns C, Stukenborg JB, Paluch-Shimon S, Halaska MJ, Uzan C, Meissner J, von Wolff M, Anderson RA, Jordan K, Committee EG (2020). Fertility preservation and post-treatment pregnancies in post-pubertal cancer patients: ESMO clinical practice guidelines. Ann Oncol.

[10] Jacobson MH, Mertens AC, Spencer JB, Manatunga AK, Howards PP (2016). Menses resumption after cancer treatment-induced amenorrhea occurs early or not at all. Fertil Steril.

[11] Lambertini M, Moore HCF, Leonard RCF, Loibl S, Munster P, Bruzzone M, Boni L, Unger JM, Anderson RA, Mehta K, Minton S, Poggio F, Albain KS, Adamson DJA, Gerber B, Cripps A, Bertelli G, Seiler S, Ceppi M, Partridge AH, Del Mastro L (2018). Gonadotropin-releasing hormone agonists during chemotherapy for preservation of ovarian function and fertility in premenopausal patients with early breast cancer: a systematic review and meta-analysis of individual patient-level data. J Clin Oncol.

[12] Lambertini M, Blondeaux E, Perrone F, Del Mastro L (2020). Improving adjuvant endocrine treatment tailoring in premenopausal women with hormone receptor-positive breast cancer. J Clin Oncol.

[13] Dewailly D, Andersen CY, Balen A, Broekmans F, Dilaver N, Fanchin R, Griesinger G, Kelsey TW, La Marca A, Lambalk C, Mason H, Nelson SM, Visser JA, Wallace WH, Anderson RA (2014). The physiology and clinical utility of anti-mullerian hormone in women. Hum Reprod Update.

[14] Finkelstein JS, Lee H, Karlamangla A, Neer RM, Sluss PM, Burnett-Bowie SM, Darakananda K, Donahoe PK, Harlow SD, Prizand SH, Joffe H, Kumar A, Martin DE, McConnell D, Merrilat S, Morrison A, Pastore LM, Randolph JF, Greendale GA, Santoro N (2020). Antimullerian hormone and impending menopause in late reproductive age: the study of women’s health across the nation. J Clin Endocrinol Metab.

[15] Kelsey TW, Wright P, Nelson SM, Anderson RA, Wallace WH (2011). A validated model of serum anti-müllerian hormone from conception to menopause. PLoS ONE.

[16] Anderson RA, Su HI (2020). The clinical value and interpretation of anti-mullerian hormone in women with cancer. Front Endocrinol (Lausanne).

[17] Anderson RA, Themmen APN, Al Qahtani A, Groome NP, Cameron DA (2006). The effects of chemotherapy and long-term gonadotrophin suppression on the ovarian reserve in premenopausal women with breast cancer. Hum Reprod.

[18] Dezellus A, Barriere P, Campone M, Lemanski C, Vanlemmens L, Mignot L, Delozier T, Levy C, Bendavid C, Debled M, Bachelot T, Jouannaud C, Loustalot C, Mouret-Reynier MA, Gallais-Umbert A, Masson D, Freour T (2017). Prospective evaluation of serum anti-mullerian hormone dynamics in 250 women of reproductive age treated with chemotherapy for breast cancer. Eur J Cancer.

[19] Freour T, Barriere P, Masson D (2017). Anti-mullerian hormone levels and evolution in women of reproductive age with breast cancer treated with chemotherapy. Eur J Cancer.

[20] Lambertini M, Olympios N, Lequesne J, Calbrix C, Fontanilles M, Loeb A, Leheurteur M, Demeestere I, Di Fiore F, Perdrix A, Clatot F (2019). Impact of taxanes, endocrine therapy, and deleterious germline BRCA mutations on anti-mullerian hormone levels in early breast cancer patients treated with anthracycline- and cyclophosphamide-based chemotherapy. Front Oncol.

[21] Anderson RA, Cameron DA (2011). Pretreatment serum anti-mullerian hormone predicts long-term ovarian function and bone mass after chemotherapy for early breast cancer. J Clin Endocrinol Metab.

[22] Anderson RA, Rosendahl M, Kelsey TW, Cameron DA (2013). Pretreatment anti-mullerian hormone predicts for loss of ovarian function after chemotherapy for early breast cancer. Eur J Cancer.

[23] Barnabei A, Strigari L, Marchetti P, Sini V, De Vecchis L, Corsello SM, Torino F (2015). Predicting ovarian activity in women affected by early breast cancer: a meta-analysis-based nomogram. Oncologist.

[24] Ruddy KJ, O'Neill A, Miller KD, Schneider BP, Baker E, Sparano JA, Dang C, Northfelt DW, Sledge GW, Partridge AH (2014). Biomarker prediction of chemotherapy-related amenorrhea in premenopausal women with breast cancer participating in E5103. Breast Cancer Res Treat.

[25] Xue C, Wei W, Sun P, Zheng W, Diao X, Xu F, Huang J, An X, Xia W, Hong R, Jiang K, Huang R, Yuan Z, Wang S, Li A, Zou R, Shi Y (2019). Pretreatment anti-mullerian hormone-based nomogram predicts menstruation status after chemotherapy for premenopausal women with hormone receptor-positive early breast cancer. Breast Cancer Res Treat.

[26] Su HC, Haunschild C, Chung K, Komrokian S, Boles S, Sammel MD, DeMichele A (2014). Prechemotherapy antimullerian hormone, age, and body size predict timing of return of ovarian function in young breast cancer patients. Cancer.

[27] Anderson RA, Mansi J, Coleman RE, Adamson DJA, Leonard RCF (2017). The utility of anti-mullerian hormone in the diagnosis and prediction of loss of ovarian function following chemotherapy for early breast cancer. Eur J Cancer.

[28] Chai J, Howie AF, Cameron DA, Anderson RA (2014). A highly-sensitive anti-mullerian hormone assay improves analysis of ovarian function following chemotherapy for early breast cancer. Eur J Cancer.

[29] Kim HA, Choi J, Park CS, Seong MK, Hong SE, Kim JS, Park IC, Lee JK, Noh WC, Ati T (2018). Post-chemotherapy serum anti-mullerian hormone level predicts ovarian function recovery. Endocr Connect.

[30] De Vos FY, van Laarhoven HW, Laven JS, Themmen AP, Beex LV, Sweep CG, Seynaeve C, Jager A (2012). Menopausal status and adjuvant hormonal therapy for breast cancer patients: a practical guideline. Crit Rev Oncol Hematol.

[31] Rosner W, Hankinson SE, Sluss PM, Vesper HW, Wierman ME (2013). Challenges to the measurement of estradiol: an endocrine society position statement. J Clin Endocrinol Metab.

[32] Bertelsen BE, Kellmann R, Viste K, Bjornevik AT, Eikesdal HP, Lonning PE, Sagen JV, Almas B (2020). An ultrasensitive routine LC-MS/MS method for estradiol and estrone in the clinically relevant sub-picomolar range. J Endocr Soc.

[33] Kim HA, Lee JW, Nam SJ, Park BW, Im SA, Lee ES, Jung YS, Yoon JH, Kang SS, Lee SJ, Park KH, Jeong J, Cho SH, Kim SY, Kim LS, Moon BI, Lee MH, Kim TH, Park C, Jung SH, Gwak G, Kim J, Kang SH, Jin YW, Kim HJ, Han SH, Han W, Hur MH, Noh WC, Korean Breast Cancer Study G (2020). Adding ovarian suppression to tamoxifen for premenopausal breast cancer: a randomized phase III trial. J Clin Oncol.

[34] Webber L, Davies M, Anderson R, Bartlett J, Braat D, Cartwright B, Cifkova R, de Muinck K-S, Hogervorst E, Janse F, Liao L, Vlaisavljevic V, Zillikens C, Vermeulen N, ESHRE Guideline Group on POI (2016). ESHRE guideline: management of women with premature ovarian insufficiency. Hum Reprod.

[35] Harlow SD, Gass M, Hall JE, Lobo R, Maki P, Rebar RW, Sherman S, Sluss PM, de Villiers TJ, Group SC (2012). Executive summary of the stages of reproductive aging workshop + 10: addressing the unfinished agenda of staging reproductive aging. J Clin Endocrinol Metab.

[36] Panay N, Anderson RA, Nappi RE, Vincent AJ, Vujovic S, Webber L, Wolfman W (2020). Premature ovarian insufficiency: an International Menopause Society White Paper. Climacteric.

